# Enzymatic and quantitative properties of Rubisco in C_3_ herbaceous plants with early-spring persistent leaves and some alpine plants

**DOI:** 10.1007/s10265-026-01701-8

**Published:** 2026-03-23

**Authors:** Sakiko Sugawara, Kana Ito, Shin-Ichi Miyazawa, Amane Makino, Yuji Suzuki

**Affiliations:** 1 Graduate School of Arts and Sciences, Iwate University, Morioka, Japan; 2 Forestry and Forest Products Research Institute, Tsukuba, Japan; 3 Graduate School of Agricultural Science, Tohoku University, Sendai, Japan; 4 Faculty of Agriculture, Iwate University, 3-18-8 Ueda, Morioka, 020-8550 Japan; 5 Present Address: Institute for Excellence in Higher Education, Tohoku University, Sendai, 980-8576 Japan

**Keywords:** Carboxylase activity, Low temperature, N allocation, Plant evolution, Rubisco

## Abstract

Plants adapted to cool habitats have been suggested to possess Rubisco with a high carboxylation capacity and low CO_2_ affinity, thereby facilitating efficient photosynthesis at low temperatures. Rubisco with a low CO_2_ affinity is believed to be less abundant owing to its concomitant high carboxylation capacity. This study aimed to provide information on these subjects by examining plants with early-spring persistent leaves and alpine plants. In these plants, the turnover rates of Rubisco carboxylation under CO_2_-saturated conditions (*k*_cat_^c^) were low to moderate, similar to those of the control C_3_ crops. The predicted Rubisco CO_2_ affinity, that is, the ratio of Rubisco carboxylase activity under CO_2_-unsaturated conditions (*v*_cu_), measured using reaction mixtures saturated with N_2_ gas containing CO_2_ and O_2_ at present atmospheric levels to *k*_cat_^c^, tended to be moderate, as observed in the control C_3_ crops. Some of these plants contained Rubisco with a low *k*_cat_^c^ and moderate CO_2_ affinity, although Rubisco with a low *k*_cat_^c^ was reported to exhibit a high CO_2_ affinity. N allocation to Rubisco in these plants was comparable to or slightly lower than that in control C_3_ crops. No significant correlations were observed between N allocation to Rubisco and *k*_cat_^c^ or predicted Rubisco CO_2_ affinity. These results suggest that, for plants with early-spring persistent leaves and alpine plants, Rubisco kinetics is not necessarily advantageous for photosynthesis at low temperatures, and that Rubisco abundance is not necessarily dependent on Rubisco kinetics. The physiological implications of these phenomena are discussed.

**Supplementary Information:**

The online version contains supplementary material available at 10.1007/s10265-026-01701-8.

## Introduction

Rubisco (EC 4. 1. 1. 39) catalyzes the fixation of CO_2_ to ribulose 1,5-bisphosphate (RuBP), resulting in the formation of 3-phosphoglycerate (3-PGA) in the first reaction of the Calvin–Benson cycle. The CO_2_ fixation by Rubisco is a slow reaction, with average turnover numbers (*k*_cat_^c^) of 3.3 and 4.4 s^− 1^ in C_3_ and C_4_ plants, respectively (Flamholz et al. 2019). Rubisco also catalyzes the oxygenation of RuBP and produces 2-phosphoglycolate in the presence of O_2_, which is subsequently detoxified through photorespiration, a complex, energy-consuming, and CO_2_-releasing metabolic pathway (Ogren 1984). Natural variations in Rubisco kinetics exist and are believed to be influenced by the specific environmental conditions to which the plants have adapted. For example, C_3_ plants that are adapted to cool habitats typically exhibit higher *k*_cat_^c^ values than those adapted to warm habitats (Ishikawa et al. 2009; Sage 2002). The *k*_cat_^c^ and CO_2_ affinity of Rubisco tend to have a trade-off relationship among various photosynthetic organisms (Flamholz et al. 2019 and the references therein). This relationship suggests that the CO_2_ affinity of Rubisco in C_3_ plants adapted to cool habitats tends to be lower than in C_3_ plants adapted to warm habitats. These Rubisco characteristics are suggested to result from its molecular evolution to maximize photosynthesis. In cool habitats, Rubisco with high *k*_cat_^c^ is believed to be advantageous for photosynthesis despite its low CO_2_ affinity, as O_2_ solubility in water is high at low temperatures (Sage 2002). In contrast, the relationship between Rubisco kinetics and climate may not be generalizable among C_3_ crops (Hermida-Carrera et al. 2016) or various plant species from diverse climates (Orr et al. 2016), implying that the effects of artificial selection of crops or other climatic factors are not negligible. In addition, Rubisco abundance has been suggested to correlate with Rubisco kinetics for efficient photosynthesis. For example, among various plant species, Rubisco/total protein ratios tend to be high for Rubisco with a high CO_2_ affinity to compensate for the concomitant low carboxylation capacity (Galmés et al. 2014). This result also means that Rubisco with a low CO_2_ affinity and high *k*_cat_^c^ is less abundant, as its high carboxylation capacity reduces the requirement for increased abundance. Similar trends were observed between N allocation to Rubisco and the approximately predicted Rubisco CO_2_ affinity among C_3_ and C_4_ crops and conifers, although exceptions include lycopods harboring with very small quantities of Rubisco and Equisetales containing abundant Rubisco with low CO_2_ affinity (Ito et al. 2024; Sugawara et al. 2025). If Rubisco kinetics showed adaptation to low temperatures, this trend in the kinetics and abundance of Rubisco would be evident in plants adapted to cool habitats. In the present study, we aimed to contribute additional insights on these subjects by investigating plants whose leaves are persistent in early spring and alpine plants, which are exposed to notably low temperatures. Consequently, *k*_cat_^c^, the approximate Rubisco CO_2_ affinity predicted from the ratio of Rubisco carboxylation activity under CO_2_-unsaturated conditions per Rubisco active site (*v*_cu_) to *k*_cat_^c^, and N allocation to Rubisco were determined using methods developed for recalcitrant plant species (Ito et al. 2024; Sugawara et al. 2025), and their relationships were analyzed.

## Materials and methods

### Plant materials and sampling

Sampling of plant species with early-spring persistent leaves was conducted of the Iwate University campus located in Morioka, Japan (39º4′ N, 141º1′ E, 130 m latitude, Andisol soil type) between March and May in 2024. In Morioka, the average maximum day temperatures in March, April, and May 2024 were 7.3 °C, 19.9 °C, and 22.0 °C, respectively, according to the AMeDAS data collected by the Japan Meteorological Agency, whereas the minimum temperatures were − 1.6 °C, 6.7 °C, and 9.7 °C. In April 2024, the average maximum and minimum day temperatures were higher than the 30-year-average values by 5.5 and 3.5 °C, respectively, whereas differences between the other temperatures in 2024 and 30-year-average values were ˂ 2 °C. The plant species sampled are listed below. The flowering periods in Morioka in 1996 (Suda and Hasegawa 1999) or in the neighboring Shiwa town (Suda and Shirawawa 1996; the periods of the survey is not specified) is shown in parentheses. Biennial herbaceous species included red dead nettle (*Lamium purpureum* L.) (late March to late May) and annual honesty (*Lunaria annua* L.) (mid-May to early June). Perennial herbaceous species included crocus (*Crocus vernus* L.) (early April to late May), adonis (*Adonis ramosae* Franch.) (mid-March to late April), dandelion (*Taraxacum officinale* ex F.H. Wigg.) (mid-April to mid-June and mid-August and early November), comfrey (*Symphytum officinale* L.) (early June to late June), and spiderwort (*Tradescantia virginiana* L.) (not reported in these studies; mid-June to mid-August for the related species *T. ohiensis* Raf.). Among these plant species, *C. vernus* and *A. ramosae* exhibit typical spring-ephemeral traits, completing their life cycles by early summer (Kudo 1995; Lapointe 2001). Evergreen herbaceous species included lenten rose (*Helleborus orientalis* Lam.) (mid-April to mid-May) and bigleaf periwinkle (*Vinca major* L.) (early May to mid-June). *Helleborus* plants are cold-tolerant and heat-sensitive (Lowder et al. 2010). *V. major* is also cold-tolerant but can grow in summer (Seyedabadi et al. 2021). The biennial and perennial plants used have emerged during the first half of April at the latest. Sampling was conducted when the plants were vigorously growing before flowering or while plants were vigorous after flowering. The sampling dates are shown in Table 1. The leaves from these plants were exercised from different individuals, and immediately floated on water in a Petri dish. Subsequently, they were transferred to the laboratory and placed in a small air-conditioned growth chamber (LH-60FL 12-DT, NK system) equipped with a 300 W light-emitting diode lamp unit for plant culture (UPA300, Shenzhen Uplighting Technology, Shenzhen, China). The chamber was operated at a photosynthetic photon flux density (PPFD) of 680 µmol m^−2^ s^−1^ and an air temperature of 25 °C. Following exposure to these conditions for a minimum of 30 min, leaves were immediately used for the Rubisco assay.

**Table 1:** Turnover rates of Rubisco carboxylation (*k*_cat_^c^), percentages of Rubisco carboxylation activity at unsaturated CO_2_ conditions in *k*_cat_^c^ (*v*_cu_/*k*_cat_^c^), total leaf-N and Rubisco levels, percentages of Rubisco-N in total leaf-N (Rubisco-N), and sampling dates in the control C_3_ crops, plants with early-spring persistent leaves, and alpine plants^1^

	*k*_cat_^c^ (s^− 1^)	*v*_cu_*/k*_cat_^c^ (%)	*N* (mg g^− 1^ FW)	Rubisco (mg g^− 1^ FW)	Rubisco-*N* (%)	Sampling date
Control plants						
*Oryza sativa*	1.18 ± 0.03^df^	68.3 ± 1.5^a^	11.0 ± 0.4^a^	20.1 ± 1.1^a^	29.3 ± 0.9^ab^	–^2^
*Hordeum vulgare*	1.48 ± 0.08 ^bc^	56.1 ± 1.6^bc^	6.0 ± 0.2^cd^	10.1 ± 1.0^cde^	26.8 ± 1.7^ac^	–
*Spinacia oleracea*	1.83 ± 0.11^a^	54.6 ± 2.4^bd^	4.9 ± 0.5^de^	7.7 ± 1.5^dg^	24.7 ± 2.9^bcd^	–
Spring plants						
*Lamium purpureum*	0.96 ± 0.06^f^	54.5 ± 2.8^bd^	5.5 ± 0.1^ce^	8.5 ± 0.2^df^	24.8 ± 0.8^bcd^	April 15 and 16^3^
*Symphytum officinale*	1.05 ± 0.01^ef^	51.4 ± 1.0^cd^	NA^4^	NA	NA	May 20
*Adonis ramose*	1.22 ± 0.04^cdf^	63.1 ± 0.3^ab^	7.7 ± 0.2^b^	11.9 ± 0.4^c^	24.8 ± 0.8^bcd^	April 3 and 10
*Crocus vernus*	1.23 ± 0.06^cde^	65.2 ± 3.6^a^	10.1 ± 0.2^a^	15.6 ± 0.4^b^	24.8 ± 1.1^bcd^	March 26 and 28
*Tradescantia virginiana*	1.23 ± 0.04^cde^	50.1 ± 0.9^cd^	5.4 ± 0.4^ce^	10.7 ± 1.1^cd^	31.8 ± 2.9^a^	May 20 and 23
*Lunaria annua*	1.32 ± 0.01^bd^	68.8 ± 1.3^a^	5.8 ± 0.7^cd^	9.7 ± 1.1^cde^	26.7 ± 0.8^ac^	April 22 and 25
*Helleborus orientalis*	1.43 ± 0.05^bd^	52.6 ± 1.1^cd^	5.9 ± 0.7^cd^	7.8 ± 1.7^dg^	20.8 ± 2.7^ce^	April 9 and 10
*Taraxacum officinale*	1.50 ± 0.03^b^	55.2 ± 3.2^bd^	6.7 ± 0.0^bc^	10.8 ± 0.2^cd^	25.9 ± 0.5^ac^	April 15,16 and 22
*Vinca major*	1.80 ± 0.09^a^	51.2 ± 0.6^cd^	5.8 ± 0.1^cd^	7.1 ± 0.3^efg^	19.5 ± 0.8^de^	May 27 and 30
Alpine plants						
*Schoenoplectus hondoensis*	1.18 ± 0.04^df^	51.6 ± 5.9^cd^	4.4 ± 0.2^de^	6.4 ± 0.5^fg^	23.0 ± 1.0^ce^	September 20^3^
*Trichophorum cespitosum*	1.28 ± 0.17^bde^	54.2 ± 3.2^bd^	5.5 ± 0.4^ce^	5.7 ± 0.7^fg^	16.6 ± 2.6^e^	September 20
*Dicentra peregrina*	1.82 ± 0.08^a^	47.3 ± 1.4^d^	4.2 ± 0.8^e^	5.4 ± 0.9^g^	20.3 ± 1.4^ce^	June 20

^1^Data are presented as mean ± SD (*n* = 3). Analysis of variance was performed for all samples, followed by the Tukey–Kramer test. Symbols with the same letter are not significantly different (*p* < 0.05)

^2^Plant culture and sampling were conducted under controlled environments

^3^Sampling for alpine plants and plants with early-spring persistent leaves was conducted in 2023 and 2024, respectively

^4^Data were not available because of the extreme sample viscosity

Komakusa (*Dicentra peregrina* (Rudolph) Makino), *Schoenoplectiella hondoensis* (Ohwi) Hayas., and *Trichophorum cespitosum* (L.) Hartm. were used as the alpine plants. These are perennial herbaceous plants. *D. peregrina* plants were collected from gravelly areas in Mt. Akita-Komagatake, located in Shizukuishi-cho, Iwate Prefecture, Japan (39°8′ N, 140°8′ E, 1,450 m latitude, immature soil type) on June 20, 2023, just before the timing of flowering onset in July (Inawashiro and Katayama 1991). Different individuals were collected from the soil, transferred to the laboratory, and maintained in a growth chamber (NC-411HC, NK system, Osaka, Japan) equipped with a light-emitting diode (LED) light unit (PFQ-600DT, NK system). Daytime ranged from 0500 to 1900. All fluorescent lights were turned on during the day, whereas the LED lights were turned on from 0815 to 1545. The maximum light intensity during daytime was a PPFD of 500 µmol quanta m^− 2^ s^−1^. The day/night temperature regimes were 16 °C from 0500 to 0800, 18 °C from 0800 to 1600, 16 °C from 1600 to 1900, and 14 °C at night. CO_2_ level and relative humidity were adjusted to 400 ppm and 50%, respectively. The plants were irrigated occasionally using tap water. Leaves were excised at approximately 1300 and immediately used for the Rubisco assay. Leaves of *S. hondoensis* and *T. cespitosum* were collected from different individuals grown in wetlands in Hachimantai, located in Iwate Prefecture (40°0′ N, 140°9′ E, 1,600 m latitude, Podzol soil type) on September 20, 2023, while these plants were vigorous after the time of flowering onset in July (Inawashiro and Katayama 1991; Kudo 1978). Leaves exposed to direct sunlight were excised at approximately 1200, immediately frozen in liquid N_2_, transferred to the laboratory, and used for the Rubisco assay. The meteorological data for these areas were not available.

Rice (*Oryza sativa* L.) plants were grown hydroponically (Makino et al. 1988) in an air-conditioned greenhouse with supplemental light provided by an LED lamp unit (PFQ-600DT, NK system, Osaka, Japan) at a PPFD of 1,000 µmol quanta m^−2^ s^−1^ at plant height (Ito et al. 2024). The day and night temperatures were 25 °C and 20 °C, respectively. Spinach (*Spinacia oleracea* L.) plants were grown in the same greenhouse using a commercial culture soil. Barley (*Hordeum vulgare* L.) plants were grown in a greenhouse with the air temperature continuously maintained at 25 °C, using commercial culture soil, and were transferred to the greenhouse used for the cultivation of *O. sativa* and *S. oleracea* prior to sampling. These plants were exposed to LED supplemental light between 1100 and 1300 for at least 30 min. The leaves were immediately frozen in liquid N_2_ and used for the Rubisco assay.

Leaves equivalent to those used for the Rubisco assay were used for total leaf-N and Rubisco determination, except that *O. sativa* plants were grown under similar air temperature and light intensity conditions in an environmentally controlled growth chamber (NC-411HC, NK system). Leaves were collected, weighed, and immediately used or frozen in liquid N_2_ and stored at − 80 °C until use.

### Rubisco assay

The Rubisco assay was performed as previously described (Sugawara et al. 2025). Briefly, leaf samples were quickly homogenized in an extraction buffer [200 mM HEPES-NaOH (pH 8.0) containing 50 mM 2-mercaptoethanol, 20 mM MgCl_2_, 10 mM dithiothreitol, 1 mM Na_2_HPO_4_, 1 mM EDTA, 12.5% (v/v) glycerol, 6% (w/v) polyethylene glycol 4000, and 5% (w/v) polyvinylpolypyrrolidone], followed by centrifugation. Rubisco in the supernatant was pelleted after the addition of polyethylene glycol 4000, followed by centrifugation (Hall and Tolbert 1978). The Rubisco-containing pellet was dissolved in a buffer and desalted. An aliquot of the resulting sample solution was used for spectrophotometric measurement of Rubisco carboxylase activity under CO_2_-saturated conditions in the presence of 10 mM NaHCO_3_ in the reaction mixture by coupling 3-PGA formation with NADH oxidation at 25 °C according to the Lilley and Walker (1974) method, with slight modifications (Sugawara et al. 2025). Rubisco carboxylase activity under CO_2_-unsaturated conditions was measured using another aliquot of the sample solution by the Lilley and Walker method, except that a bicarbonate-free reaction mixture equilibrated with a N_2_ gas containing 21% O_2_ and 396.0 ppm CO_2_ at 25 °C was used instead of a CO_2_-saturated reaction mixture. The remaining sample solution was supplemented with monoiodoacetic acid and 2-mercaptoethanol, heat-treated with sodium dodecyl sulfate (SDS), and stored at − 30 °C until Rubisco determination. *v*_cu_ and *k*_cat_^c^ were calculated by dividing the CO_2_-unsaturated and CO_2_-satureted Rubisco carboxylase activities, respectively, by the number of Rubisco catalytic sites in the sample used for the assay. The percentage of *v*_cu_ in *k*_cat_^c^ was used to predict the CO_2_ affinity of Rubisco.

### Rubisco and total leaf-N determination

Rubisco and total leaf-N were determined as previously described (Sugawara et al. 2025). Briefly, the leaf samples were ground to a fine powder with acid-washed quartz sand using liquid N_2_. The sample powder was added to the sodium phosphate buffer and immediately mixed. A portion of the leaf homogenate was used to determine total leaf-N level. The remaining portion was homogenized after the addition of polyvinylpyrrolidone 25, heat-treated with SDS, and centrifuged. The resulting supernatant was kept at − 30 °C until Rubisco determination. The homogenate for total-N determination was subjected to Kjeldahl digestion, followed by N determination using Nessler’s reagent, as described by Makino et al. (1984). Rubisco levels were determined by formamide extraction of Coomassie Brilliant Blue R-250-stained bands corresponding to the large and small subunits of Rubisco separated by SDS-polyacrylamide gel electrophoresis (Makino et al. 1985; Suzuki et al. 2023). The amino acid sequences of the Rubisco large subunit of the plant species used in the present study which are available in public databases were highly homologous (Fig. S1), although those of the Rubisco small subunit are not available in public databases. Therefore, differences in Rubisco amino acid sequences would not notably affect Rubisco abundance or *k*_cat_^c^.

### Statistical analysis

Statistical analyses were conducted using analysis of variance, followed by the Tukey–Kramer test, with R (version 4.1.3; Ihaka and Gentleman 1996) and the multicomp package (version 1.4-8; Hothorn et al. 2008).

## Results

Among the control C_3_ crops, the *k*_cat_^c^ values in *O. sativa* were the lowest, whereas those in *S. oleracea* were the highest, approximately 1.5-fold higher than those in *O. sativa* (Table 1). The *k*_cat_^c^ values of *H. vulgare* were intermediate between those of *O. sativa* and *S. oleracea*. The relative relationships were similar to those previously reported (see the aggregated data in Flamholz et al. 2019). These *k*_cat_^c^ values were lower than those determined using radioisotope methods, as expected from previous studies (Reid et al. 1997). Some variations in *k*_cat_^c^ values were observed among the plants with early-spring persistent leaves and alpine plants. *k*_cat_^c^ values of several plants were comparable to those of *O. sativa*. The lowest values obtained for *L. purpureum* were lower than the *O. sativa k*_cat_^c^ values. The highest values obtained for *V. major* did not exceed the *S. oleracea k*_cat_^c^ values.

*v*_cu_/*k*_cat_^c^ percentages were higher in *O. sativa* than in *H. vulgare* and *S. oleracea* among the control C_3_ crops (Table 1). Variations in the *v*_cu_/*k*_cat_^c^ percentage in plants with early-spring persistent leaves and alpine plants tended to be smaller than those in the *k*_cat_^c^ value. Most of the *v*_cu_/*k*_cat_^c^ percentages were comparable to those in *H. vulgare* and *S. oleracea*, except that the *v*_cu_/*k*_cat_^c^ percentages in *C. vernus*, *A. ramosae*, and *L. annua* were comparable to those in *O. sativa*.

Total leaf-N and Rubisco levels were the highest in *O. sativa* and lowest in *S. oleracea* among the control C_3_ crops (Table 1). Some variations in total leaf-N and Rubisco levels were observed among plants with early-spring persistent leaves. Most values were intermediate between those of *O. sativa* and *S. oleracea*. In contrast, the total leaf-N or Rubisco levels in alpine plants tended to be lower than those in *S. oleracea*. Total leaf-N and Rubisco levels in *S. officinale* were difficult to determine because of the substantial viscosity of the homogenates.

The Rubisco-N percentages in total leaf-N were the highest in *O. sativa* and lowest in *S. oleracea* among the control C_3_ crops (Table 1). Several plants with early-spring persistent leaves had Rubisco-N percentages comparable to those of *S. oleracea* and *H. vulgare*, whereas the Rubisco-N percentage of *T. virginiana* tended to be higher than those in *O. sativa*. In *H. orientalis* and *V. major*, which are evergreen herbaceous plants, and in alpine plants, Rubisco-N percentages were lower than those in *S. oleracea*.

**Fig. 1:**
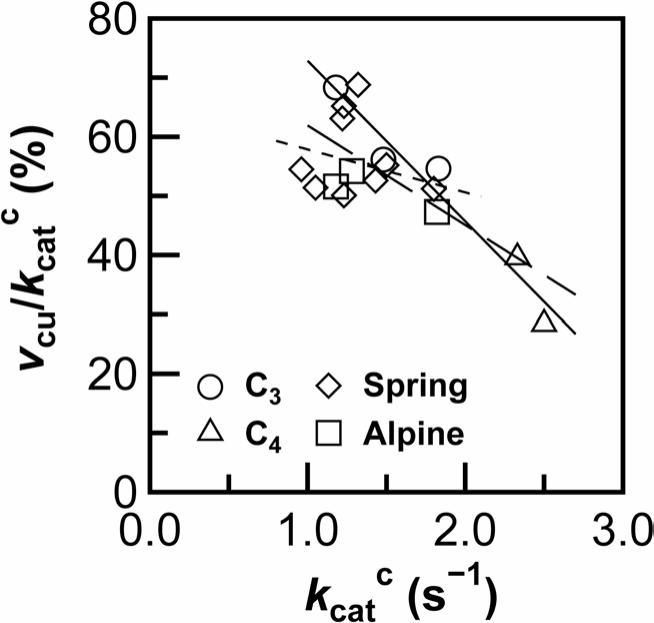
Relationship between *v*_cu_*/k*_cat_^c^, which represents the percentage of Rubisco carboxylase activity at unsaturated CO_2_ conditions (*v*_cu_) in turnover rate of Rubisco carboxylase activity (*k*_cat_^c^), and *k*_cat_^c^ in the control C_3_ and C_4_ crops, plants with early-spring persistent leaves (Spring), and alpine plants (Alpine). The average values presented in Table 1 are used, except those for *Z. mays* and *S. bicolor*, which are sourced from Ito et al. (2024). Circles, triangles, diamonds, and squares represent C_3_ crops, C_4_ crops, plants with early-spring persistent leaves, and alpine plants, respectively. The solid, dashed, and dotted lines represents regression lines for the control C_3_ and C_4_ crops [*y* = − 2.71 ×1×0^1^*x* + 9.99 × 10^1^, *R* = − 0.972 (*p* = 0.001)], all plants [*y* = − 1.68 × 10^1^*x* + 7.87 × 10^1^, *R* = − 0.734 (*p* = 0.001)], and plants with early-spring persistent leaves and alpine plants [*y* = − 7.26 *x* + 6.51 × 10^1^, *R* = − 0.289 (*p* = 0.358)], respectively

The relationships among the Rubisco parameters were examined. The values reported for maize (*Zea mays*) and sorghum (*Sorghum bicolor*) by Ito et al. (2024) served as the control C_4_ crops. A strong negative correlation was observed between the *v*_cu_/*k*_cat_^c^ percentages and *k*_cat_^c^ values among the control crops (Fig. 1). A negative correlation was also observed when all data were included in the analysis. These results are consistent with the general trade-off relationship between *k*_cat_^c^ and CO_2_ affinity (Flamholz et al. 2019). However, when the data obtained from plants with early-spring persistent leaves and from alpine plants were analyzed separately, no significant correlation was observed. Approximately half of the data points obtained from these plants were positioned around the regression line for the control crops. The remaining data points tended to be positioned below this regression line, indicating that the *v*_cu_/*k*_cat_^c^ values were lower than expected based on the *k*_cat_^c^ values.

**Fig. 2:**
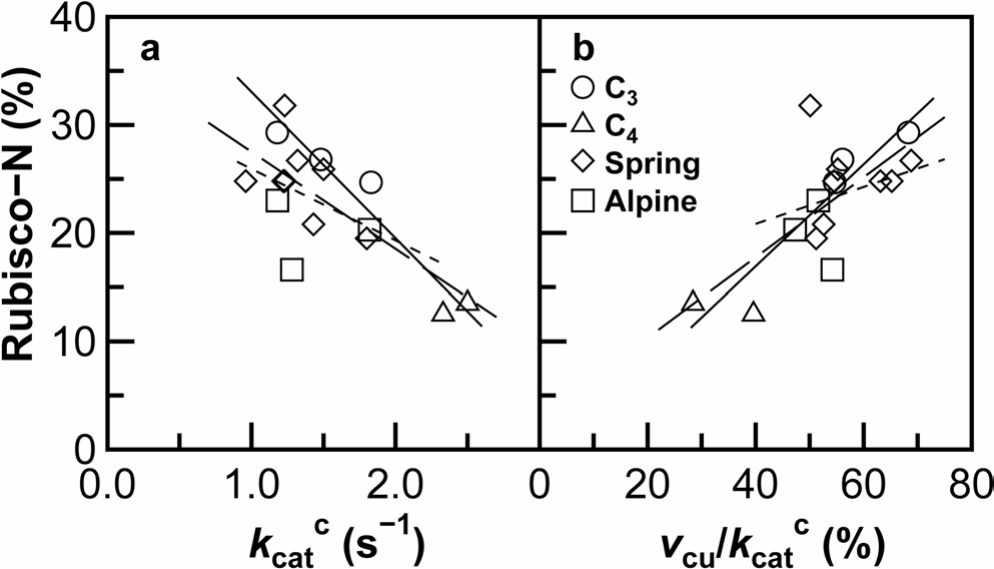
Relationship between the percentage of N allocated to Rubisco in total leaf-N (Rubisco-N) and the turnover rate of Rubisco carboxylation (*k*_cat_^c^) (**a**), or *v*_cu_*/k*_cat_^c^, which represents the percentage of Rubisco carboxylase activity at unsaturated CO_2_ conditions (*v*_cu_) in *k*_cat_^c^ (**b**), in the control C_3_ and C_4_ crops, plants with early-spring persistent leaves, and alpine plants. The average values presented in Table 1 are used, except those for *Z. mays* and *S. bicolor*, which are sourced from Ito et al. (2024). Symbols and regression lines are the same as those shown in Fig. 1. The formulae for the regression lines are as follows: *y* = − 1.34 × 10^1^ *x* + 4.63 × 10^1^, *R* = − 0.963 (*p* = 0.002) in (**a**) and *y* = 4.69 × 10^–1^ *x* – 1.85, *R* = 0.938 (*p* = 0.005) in (**b**) for control C_3_ and C_4_ crops, *y* = − 8.95 *x* + 3.64 × 10^1^, *R* = − 0.722 (*p* = 0.001) in (**a**) and *y* = 3.69 × 10^–1^ *x* + 2.97, *R* = 0.707 (*p* = 0.002) in (**b**) for all plants, and *y* = − 6.62 *x* + 3.25 × 10^1^, *R* = − 0.418 (*p* = 0.195) in (**a**) and *y* = 1.71 × 10^–1^ *x* + 1.40 × 10^–1^, *R* = 0.283 (*p* = 1.000) in (**b**) for plants with early-spring persistent leaves, and alpine plants

The Rubisco-N percentages were strongly and negatively correlated with the *k*_cat_^c^ values in the control C_3_ and C_4_ crops (Fig. 2a). A similar trend was observed when all data were included in the analysis. When the data obtained from plants with early-spring persistent leaves and from alpine plants were analyzed separately, no significant correlation was observed. Approximately half of the data points obtained from these plants were positioned around the regression line for the control crops. The others tended to be positioned below this regression line, indicating that the Rubisco-N percentages were lower than expected based on the *k*_cat_^c^ values. Rubisco-N percentages were strongly and positively correlated with *v*_cu_/*k*_cat_^c^ percentages in the control crops (Fig. 2b). A similar trend was observed when all data were included in the analysis. These results are consistent with the observation that Rubisco with high CO_2_ affinity has higher Rubisco/total protein ratios (Galmés et al. 2014). Although the data points collected from the plants with early-spring persistent leaves and alpine plants aligned with the control crop regression line, notable deviations occurred, and no significant correlation was observed.

The results for the control C_3_ crops presented in Table 1 and the correlations between the parameters presented in Figs. 1 and 2 were comparable to those in previous studies (Ito et al. 2024; Sugawara et al. 2025).

## Discussion

### Plants with early-spring persistent leaves and alpine plants do not necessarily have Rubisco with favorable kinetics for photosynthesis in cool habitats

We examined Rubisco kinetics in plants with early-spring persistent leaves and alpine plants, which are exposed to notably low temperatures. The *k*_cat_^c^ values were intermediate between those in *O. sativa* and *S. oleracea* in most plants, and lower than that in *O. sativa* in the other plants (Table 1). The *k*_cat_^c^ value in *S. oleracea* is intermediate among those reported in C_3_ plants, whereas *O. sativa* exhibits a relatively low *k*_cat_^c^ value (Flamholz et al. 2019). The approximate Rubisco CO_2_ affinity predicted from *v*_cu_/*k*_cat_^c^ percentages in most plants was comparable to those in *S. oleracea* and *H. vulgare*, both of which exhibit intermediate values of the Michaelis–Menten constant for Rubisco carboxylation (*K*c) among the C_3_ plants evaluated, and those in the other plants were comparable to those in *O. sativa*, whose *K*c value is among the lowest ones (Table 1; Flamholz et al. 2019). These results indicate that plants with early-spring persistent leaves and alpine plants do not have Rubisco with notably high *k*_cat_^c^ values and low CO_2_ affinity, which is suggested to be present in plants adapted to cold habitats and is advantageous for photosynthesis under low temperatures (Ishikawa et al. 2009; Sage 2002). These findings are consistent with those of previous studies which examined a broader range of C_3_ plant species and found no apparent relationship between Rubisco kinetics and climatic conditions (Hermida-Carrera et al. 2016; Orr et al. 2016). In addition, in some plant species, Rubisco CO_2_ affinity was not consistently high even when *k*_cat_^c^ was low, as the *v*_cu_/*k*_cat_^c^ percentages were lower than expected based on the *k*_cat_^c^ value when compared with those of the control C_3_ and C_4_ crops (Fig. 1). The results of *k*_cat_^c^ and *v*_cu_/*k*_cat_^c^ percentages suggest that Rubisco in these plant species has not evolved to optimize photosynthesis in cool habitats. *k*_cat_^c^ and CO_2_ affinity also tended to be low in plant species that diverged or diversified under elevated CO_2_ levels, such as lycopods, ferns, and conifers (Ito et al. 2024; Sugawara et al. 2025). These characteristics of Rubisco kinetics are considered unnecessary, as a control of Rubisco on photosynthesis is weak at elevated CO_2_ levels (Farquhar et al. 1980; Sharkey 1985). The results of the present study can be interpreted similarly. Photosynthetic rate at low temperatures is primarily determined not by Rubisco capacity, but by the capacities of electron transport or inorganic phosphate regeneration from sucrose or starch synthesis even when CO_2_ levels are slightly below current atmospheric concentrations (Busch and Sage 2017; Sage and Kubien 2007). In addition, C stored in underground organs contributes to growth of biennial and perennial herbaceous plants when environments are unsuitable for photosynthesis (Larcher 2001). These characteristics make Rubisco with high carboxylation capacity unnecessary in cool habitats. Therefore, Rubisco kinetics may not have been subject to strong selection pressure from low temperature conditions in these plant species.

In the present study, Rubisco assay was conducted at 25 °C, which was higher than growth temperatures of the plants with early-spring persistent leaves at their sampling times. *k*_cat_^c^ and *K*_m_ for Rubisco carboxylation generally decrease as the temperatures for Rubisco assay decreases, with some variations depending on plant species (Hermida-Carrera et al. 2016; Orr et al. 2016). When Rubisco assay was conducted in various C_3_ crops at 15 °C, 25 °C, and 35 °C, differences in the temperature dependencies of Rubisco kinetic parameters were not associated with the thermal environment of a species domestication region (Hermida-Carrera et al. 2016). Based on this observation, Rubisco in the plants with early-spring persistent leaves may also not necessarily perform well when assayed at low temperatures. This point requires further verification.

### Rubisco tends to be abundant in plants with early-spring persistent leaves except evergreen herbaceous plants, but less abundant in evergreen herbaceous plants and alpine plants

According to the Rubisco-N percentages, plants with early-spring persistent leaves, except for evergreen herbaceous plants *H. orientalis* and *V. major*, had abundant Rubisco as that in the control C_3_ crops, with *T. virginiana* containing even more abundant Rubisco (Table 1). As mentioned previously, Rubisco is not the primary factor that determines photosynthetic rate at low temperatures. However, these plant species can be exposed to warmer conditions where Rubisco tends to limit photosynthesis and abundant Rubisco contributes to increased C gain by photosynthesis. For example, temperatures transiently reach optimum levels during the growth period. Interestingly, Rubisco CO_2_ affinity in the spring ephemerals *C. vernus* and *A. ramosae* were as high as that in *O. sativa*, and their *k*_cat_^c^ values were also similar to those of *O. sativa* (Table 1). In *C. vernus* and *A. ramosae*, the combination of high Rubisco abundance and favorable kinetics may facilitate efficient photosynthesis under very brief periods of warm conditions. In addition, the plants with early-spring persistent leaves, other than the spring ephemerals, can persist beyond the spring season, according to their flowering periods (see Materials and methods). For these plant species as well, abundant Rubisco would also be advantageous for photosynthesis, for the same reasons related to Rubisco abundance, photosynthesis, and temperature conditions. Otherwise, the abundant Rubisco can serve as a storage for N nutrition (Feller et al. 2008), because its degradation products at low molecular weights are translocated to newly developing tissues as an N source (Mae et al. 1983; Makino et al. 1984). In contrast, Rubisco abundance in evergreen herbaceous plants and alpine plants tended to be low and comparable to that in conifers (Table 1; Sugawara et al. 2025). In evergreen herbaceous plants, the relatively lower abundance of Rubisco may be associated with increased leaf longevity as suggested in conifers (Sugawara et al. 2025): N allocation is suggested to shift towards cell walls rather than to the photosynthetic machinery, increasing leaf physical strength and longevity (Ghimire et al. 2017; Onoda et al. 2017). This strategy is likely adopted by evergreen herbaceous plants as well as alpine plants, which are exposed to notably stressful environments. In addition, owing to the limited availability of mineral nutrients in alpine soils, increasing N allocation to Rubisco rather than supporting leaf physical strength and longevity may not be beneficial for plant growth.

### Rubisco abundance is not closely related to Rubisco kinetics in plants with early-spring persistent leaves and alpine plants

Rubisco with high CO_2_ affinity or low *k*_cat_^c^ tended to be abundant, as previously observed when all data were included in the correlation analyses (Fig. 2; Galmés et al. 2014; Ito et al. 2024; Sugawara et al. 2025). In contrast, these relationships were not evident among plants with early-spring persistent leaves or alpine plants (Fig. 2). Notable deviations were observed compared to the relationships observed in the control C_3_ and C_4_ crops. These results suggest that Rubisco abundance is not closely related to Rubisco kinetics in plants with early-spring persistent leaves and alpine plants, despite its putative advantage for efficient photosynthesis. In plants with early-spring persistent leaves and alpine plants, Rubisco abundance is thought to be more strongly affected by the above-mentioned physiological factors.

## Concluding remarks

The present study demonstrated that plants with early-spring persistent leaves and alpine plants, which are considered to be exposed to notably low temperatures, do not necessarily have Rubisco optimized for photosynthesis at low temperatures with high *k*_cat_^c^ and low CO_2_ affinity. Certain plants have Rubisco with both low *k*_cat_^c^ and CO_2_ affinity. In these plant species, Rubisco kinetics may not have been subject to strong selection pressure from cold temperatures, as CO_2_ solubility in water is high and photosynthetic rates are not limited by Rubisco capacity at low temperature conditions. The Rubisco abundance was not closely related to Rubisco kinetics. Several factors have possibly influenced Rubisco abundance throughout evolution, including efficient photosynthesis when temperatures reach optimum levels, increased leaf longevity, and low N soil fertility. Thus, the Rubisco kinetics and abundance in these plants may not have primarily evolved for efficient photosynthesis at low temperatures. The number of alpine plants used in the present study was limited because of sampling difficulties. These conclusions should be validated using other various alpine species.

## Supplementary Information

Below is the link to the electronic supplementary material.

Supplementary Material 1

## Data Availability

The data underlying this study are available in the article and supplementary materials.
