# Supplementary material for: Enzymatic and quantitative properties of Rubisco in C3 herbaceous plants with early-spring persistent leaves and some alpine plants
Source: J Plant Res. 2026 Mar 23;139(3):511–8. doi: 10.1007/s10265-026-01701-8 (PMC13197366; doi:10.1007/s10265-026-01701-8)
Supplement: Supplementary file 1 — Supplementary Material 1 [file 10265_2026_1701_MOESM1_ESM.pdf]

Electronic supplementary materials

Title:

Enzymatic and quantitative properties of Rubisco in C_3 herbaceous plants with early-spring persistent leaves and some alpine plants

Authors:

Sakiko Sugawara[1], Kana Ito[1], Shin-Ichi Miyazawa[2], Amane Makino[3,5], Yuji Suzuki[4]

[1]Graduate School of Arts and Sciences, Iwate University, Morioka, Japan; [2]Forestry and Forest Products Research Institute, Tsukuba, Japan; [3]Graduate School of Agricultural Science, Tohoku University, Sendai, Japan; [4]Faculty of Agriculture, Iwate University, Morioka, Japan; [5]Present address: Institute for Excellence in Higher Education, Tohoku University, Sendai 980-8576, Japan.

Journal:

Journal of Plant Research

Corresponding author:

Yuji Suzuki (Faculty of Agriculture, Iwate University, 3-18-8 Ueda, Morioka 020-8550, Japan)

Tel and Fax: +81-19-621-6153

E-mail: ysuzuki@iwate-u.ac.jp

Content:

Fig. S1

```
                                   10        20        30        40        50
                          ....|....|....|....|....|....|....|....|....|....|
Oryza sativa              MSPQTETKASVGFKAGVKDYKLTYYTPEYETKDTDILAAFRVTPQPGVPP
Hordeum vulgare           .........G...Q..................................S.......
Spinacia oleracea         ...............................L..........S.......
Adonis ramosa             ..............................P....T..............
Taraxacum officinale      ...........................D......................
Lunaria annua             ..................E...............................
Symphytum officinale      ..................E...............................
Tradescantia virginiana   .............................Q....................
Vinca major               ..................E...............................

                                   60        70        80        90       100
                          ....|....|....|....|....|....|....|....|....|....|
Oryza sativa              EEAGAAVAAESSTGTWTTVWTDGLTSLDRYKGRCYHIEPVVGEDNQYIAY
Hordeum vulgare           ........................................A...S.W.C.
Spinacia oleracea         .........................N..............A..E....C.
Adonis ramosa             ........................................A..E....C.
Taraxacum officinale      ...................................G....P..ES.F...
Lunaria annua             ........................................P..ET.F...
Symphytum officinale      .....................................DA.P..E....C.
Tradescantia virginiana   ........................................P..ET.F...
Vinca major               ........................................P..ED.F...

                                  110       120       130       140       150
                          ....|....|....|....|....|....|....|....|....|....|
Oryza sativa              VAYPLDLFEEGSVTNMFTSIVGNVFGFKALRALRLEDLRIPPTYSKTFQG
Hordeum vulgare           ..................................................
Spinacia oleracea         .........................................VA.V.....
Adonis ramosa             .........................................VA.I.....
Taraxacum officinale      .........................................VA.V.....
Lunaria annua             ..............................A...........A.T.....
Symphytum officinale      .........................................TA.V.....
Tradescantia virginiana   .........................................VA.T...A.
Vinca major               ..........................................A.I.....

                                  160       170       180       190       200
                          ....|....|....|....|....|....|....|....|....|....|
Oryza sativa              PPHGIQVERDKLNKYGRPLLGCTIKPKLGLSAKNYGRACYECLRGGLDFT
Hordeum vulgare           ..................................................
Spinacia oleracea         ......................................V...........
Adonis ramosa             ......................................V...........
Taraxacum officinale      ......................................V...........
Lunaria annua             ......................................V...........
Symphytum officinale      ......A...............................V...........
Tradescantia virginiana   ......................................V...........
Vinca major               ..................................................
```

```
                                   210       220       230       240       250
                            ....|....|....|....|....|....|....|....|....|....|
Oryza sativa                KDDENVNSQPFMRWRDRFVFCAEAIYKSQAETGEIKGHYLNATAGTCEEM
Hordeum vulgare             ..................................................
Spinacia oleracea           ..................L.....L..A....................D.
Adonis ramosa               ..................L........A......................
Taraxacum officinale        ..................L......F........................
Lunaria annua               ..................L...............................
Symphytum officinale        ..................L.....L..A.................N.D..
Tradescantia virginiana     ..................L...............................
Vinca major                 ..................L........A......................

                                   260       270       280       290       300
                            ....|....|....|....|....|....|....|....|....|....|
Oryza sativa                IKRAVFARELGVPIVMHDYLTGGFTANTSLAHYCRDNGLLLHIHRAMHAV
Hordeum vulgare             ............................T.....................
Spinacia oleracea           M...........................T.S...................
Adonis ramosa               ..................................................
Taraxacum officinale        M...I.......................T.....................
Lunaria annua               ..................................................
Symphytum officinale        M...........................T..S..................
Tradescantia virginiana     L...QC.........................Y..................
Vinca major                 M...I.............................................

                                   310       320       330       340       350
                            ....|....|....|....|....|....|....|....|....|....|
Oryza sativa                IDRQKNHGMHFRVLAKALRMSGGDHIHAGTVVGKLEGEREMTLGFVDLLR
Hordeum vulgare             ...........................S......................
Spinacia oleracea           ...................L.......S...........DI.........
Adonis ramosa               ........................................I.........
Taraxacum officinale        ........I..................S............I.........
Lunaria annua               ...................L.................D..S.........
Symphytum officinale        .......................................NI.........
Tradescantia virginiana     ........................................I.........
Vinca major                 ........I..................S...........DI.........

                                   360       370       380       390       400
                            ....|....|....|....|....|....|....|....|....|....|
Oryza sativa                DDFIEKDRARGIFFTQDWVSMPGVIPVASGGIHVWHMPALTEIFGDDSVL
Hordeum vulgare             ..................................................
Spinacia oleracea           ..YT....S...Y...S...T...L.........................
Adonis ramosa               ..Y.A...S...Y.......L...L.........................
Taraxacum officinale        ........S...Y.......L...L.........................
Lunaria annua               ..YV....S...........L...L.........................
Symphytum officinale        ........T...Y.......L...L...............V.........
Tradescantia virginiana     ...V....S...............L.........................
Vinca major                 .E......S...Y.......L...L.........................
```

```
                                          410       420       430       440       450
                               ....|....|....|....|....|....|....|....|....|....|
Oryza sativa                   QFGGGTLGHPWGNAPGAAANRVALEACVQARNEGRDLAREGNEIIRSACK
Hordeum vulgare                ..............................................A...
Spinacia oleracea              .................V........................T...E.T.
Adonis ramosa                  .................V............................E...
Taraxacum officinale           .................V....................T...Q...E.T.
Lunaria annua                  .................V....................V.......E...
Symphytum officinale           .................V....V..................GD.V.E.T.
Tradescantia virginiana        .................V............................E.A.
Vinca major                    .................V....................V.......E...


                                          460       470
                               ....|....|....|....|....|....
Oryza sativa                   WSPELAAACEIWKAIKFEFEPVDKLDS--
Hordeum vulgare                ..........V............TI.KKV
Spinacia oleracea              ..........V..E.....PAM.TV----
Adonis ramosa                  ..L.......V..E......AM.T.----
Taraxacum officinale           ..........V..E......AM.TI----
Lunaria annua                  ..........V..E.R.N.PTI....GQE
Symphytum officinale           ..........V..E.T.T..E.....Y--
Tradescantia virginiana        ..........V............TV.KV-
Vinca major                    ..........V..E.T.N.KA..T.----
```

Fig. S1 Alignment of amino acid sequences of RbcL in plants used in this study, which are available from public databases. Accession numbers of RbcL proteins (GenBank for *Symphytum officinale* and *Tradescantia virginiana*, and NCBI Reference Sequence for the other plant species) are for NP_039391.1 for *Oryza sativa*, P_05698.2 for *Hordeum vulgare*, NP_054944.1 for *Spinacia oleracea*, YP_010719200.1 for *Adonis ramosae*, YP_009271390.1 for *Taraxacum officinale*, YP_009897810.1 for *Lunaria annua*, XMH33909.1 for *Symphytum officinale*, QUB02768.1 for *Tradescantia virginiana*, and YP_010437102.1 for *Vinca major*. The alignment was performed by the ClustalW algorithm (Thompson et al. (1994) Nucl Acids Res 22:4673– 4680) using BioEdit (version 7.2.5; Hall (1999) Nucl Acids Symp Ser 41:95–98) with the default parameters. Full-length amino acid sequences for the other plant species are unavailable.
